# Phase-dependent modulation of the vestibular–cerebellar network *via* combined alternating current stimulation influences human locomotion and posture

**DOI:** 10.3389/fnins.2022.1057021

**Published:** 2022-12-14

**Authors:** Hisato Nakazono, Takanori Taniguchi, Tsubasa Mitsutake, Akinori Takeda, Emi Yamada, Katsuya Ogata

**Affiliations:** ^1^Department of Occupational Therapy, Faculty of Medical Science, Fukuoka International University of Health and Welfare, Fukuoka, Japan; ^2^Department of Health Sciences, Graduate School of Medical Sciences, Kyushu University, Fukuoka, Japan; ^3^Department of Physical Therapy, Faculty of Medical Science, Fukuoka International University of Health and Welfare, Fukuoka, Japan; ^4^Research Center for Brain Communication, Research Institute, Kochi University of Technology, Kochi, Japan; ^5^Department of Linguistics, Faculty of Humanities, Kyushu University, Fukuoka, Japan; ^6^Department of Pharmaceutical Sciences, School of Pharmacy at Fukuoka, International University of Health and Welfare, Fukuoka, Japan

**Keywords:** transcranial alternating current stimulation, cerebellum, galvanic vestibular stimulation, locomotion, phase synchronization

## Abstract

**Background:**

Human locomotion induces rhythmic movements of the trunk and head. Vestibular signaling is relayed to multiple regions in the brainstem and cerebellum, and plays an essential role in maintaining head stability. However, how the vestibular–cerebellar network contributes to the rhythmic locomotor pattern in humans is unclear. Transcranial alternating current stimulation (tACS) has been used to investigate the effects of the task-related network between stimulation regions in a phase-dependent manner. Here, we investigated the relationship between the vestibular system and the cerebellum during walking imagery using combined tACS over the left cerebellum and alternating current galvanic vestibular stimulation (AC-GVS).

**Methods:**

In Experiment 1, we tested the effects of AC-GVS alone at around individual gait stride frequencies. In Experiment 2, we then determined the phase-specificity of combined stimulation at the gait frequency. Combined stimulation was applied at in-phase (0° phase lag) or anti-phase (180° phase lag) between the left vestibular and left cerebellar stimulation, and the sham stimulation. We evaluated the AC-GVS-induced periodic postural response during walking imagery or no-imagery using the peak oscillatory power on the angular velocity signals of the head in both experiments. In Experiment 2, we also examined the phase-locking value (PLV) between the periodic postural responses and the left AC-GVS signals to estimate entrainment of the postural response by AC-GVS.

**Results:**

AC-GVS alone induced the periodic postural response in the yaw and roll axes, but no interactions with imagery walking were observed in Experiment 1 (*p* > 0.05). By contrast, combined in-phase stimulation increased yaw motion (0.345 ± 0.23) compared with sham (−0.044 ± 0.19) and anti-phase stimulation (−0.066 ± 0.18) during imaginary walking (in-phase vs. other conditions, imagery: *p* < 0.05; no-imagery: *p* ≥ 0.125). Furthermore, there was a positive correlation between the yaw peak power of actual locomotion and in-phase stimulation in the imagery session (imagery: *p* = 0.041; no-imagery: *p* = 0.177). Meanwhile, we found no imagery-dependent effects in roll peak power or PLV, although in-phase stimulation enhanced roll motion and PLV in Experiment 2.

**Conclusion:**

These findings suggest that combined stimulation can influence vestibular–cerebellar network activity, and modulate postural control and locomotion systems in a temporally sensitive manner. This novel combined tACS/AC-GVS stimulation approach may advance development of therapeutic applications.

## Introduction

The cerebellar system plays a major role in balance, posture, and gait control. Postural-gait control by the cerebellum depends on sensory afferents, including vestibular information (Takakusaki, 2017). The vestibular system encodes acceleration and rotation of the head in space. Many studies have demonstrated that the vestibular system is required for the maintenance of balance and stable posture (Lopez and Blanke, 2011; Dlugaiczyk et al., 2019; Akay and Murray, 2021). However, the detailed mechanisms underlying the interaction between the vestibular system and the cerebellum with respect to effects on human locomotion remain unclear. The problem is that it is not possible to clarify whether the vestibular–cerebellar network has a functional role in generating the locomotor pattern or only maintaining balance and upright posture during walking (Akay and Murray, 2021).

Galvanic vestibular stimulation (GVS) is a non-invasive technique used to investigate sensory signal processing in the vestibular system under normal and pathological conditions (Fitzpatrick and Day, 2004; Dlugaiczyk et al., 2019). GVS induces a virtual experience of head movement and affects whole-body postural control (Fitzpatrick and Day, 2004). As alternating current GVS (AC-GVS, also called sinusoidal GVS) can produce periodic head motion (Coats, 1972; Petersen et al., 1994; Latt et al., 2003; Wuehr et al., 2018), it is useful for inducing the experience of natural head motion. Interestingly, oscillatory movements of the head and trunk are induced during gait to maintain head stability (Grossman et al., 1988; Kavanagh et al., 2005). Primarily, lateral bending and rotation of the head and trunk are governed by the gait stride frequency (referred to as the gait frequency) during normal walking (Kavanagh and Menz, 2008; van Dieën et al., 2021). Therefore, if the vestibular system plays a role in gait control, the periodic postural response induced by AC-GVS according to the gait frequency may vary between static postural control and locomotion.

**FIGURE 1:**
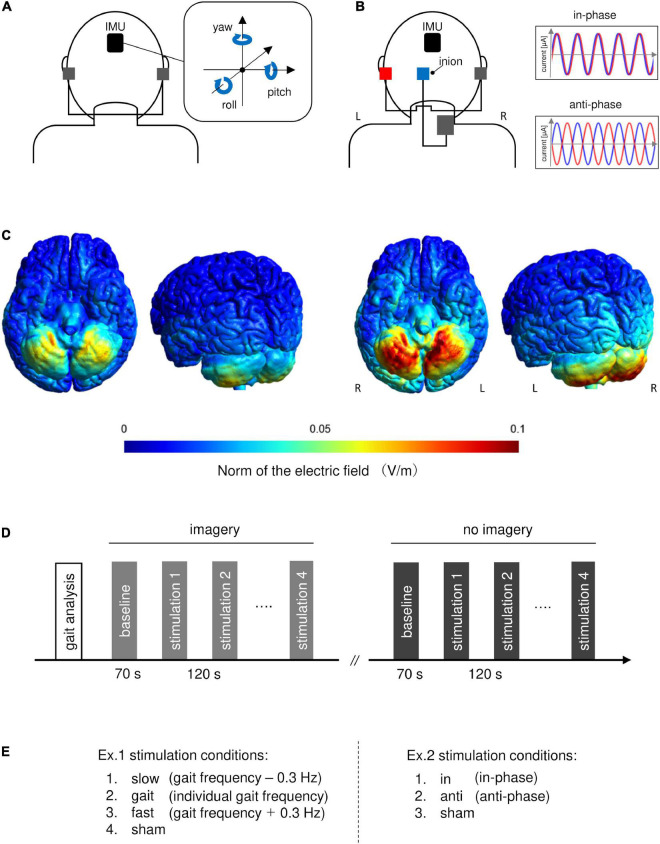
Experimental design. **(A)** Placement of AC-GVS electrodes and the axes of the inertial measurement unit (IMU). The AC-GVS electrodes were placed on the mastoid processes. We analyzed periodic head movement *via* angular velocity. **(B)** Electrode placement in the combined stimulation condition. The tACS target electrode (blue square) was centered 3 cm left-lateral from the inion. The left AC-GVS (red) and tACS of the left cerebellum (blue) were applied in two conditions: in-phase (stimulation with 0° phase difference), and anti-phase (180° phase difference). **(C)** The vector Norm of the electrical fields by AC-GVS (left two panels) or combined stimulation (right two panels). **(D)** Time course of Experiment 1. Participants underwent the two sessions (imagery and no-imagery) after gait analysis. The periodic postural response was measured under four stimulation conditions and baseline in each session. **(E)** The stimulation conditions in Experiments 1 and 2. L, left; R, right.

Transcranial alternating current stimulation (tACS) can entrain ongoing brain oscillations and modulate brain function in a frequency-dependent manner (Herrmann et al., 2013; Helfrich et al., 2014; Nakazono et al., 2020). Recently, Koganemaru et al. (2020) reported that tACS at the gait frequency over the cerebellum synchronized the gait cycle according to the tACS phase. They proposed that tACS can entrain neuronal activities related to gait generation in the cerebellum. tACS has also been used to investigate long-range functional connectivity in the brain (Polanía et al., 2012; Schwab et al., 2019; Salamanga-Giron et al., 2021). These studies applied tACS with currents that were either completely in-phase (i.e., 0° phase lag between the two regions) or anti-phase (180° phase lag between the two regions) (see Figure 1B). In-phase tACS appears to up-regulate the synchronization and connectivity between two distant regions, while anti-phase tACS seems to de-synchronize the network nodes (Bland and Sale, 2019). Therefore, “alternating current stimulation” can be utilized to probe the functional connectivity between the vestibular and cerebellar systems in terms of posture-gait control.

Here, we evaluated whether a combination of cerebellar tACS and AC-GVS could induce periodic postural responses depending on mental imagery of walking. Motor imagery experiments enable investigation of the dynamics of motor control by removing the sensory and motor complications linked to motor execution (Brinkman et al., 2016). Furthermore, functional magnetic resonance imaging (fMRI) studies have reported that locomotor regions including the cerebellum were activated during walking imagery, while the vestibular cortex was activated during imagery of standing but not imagery of walking or running (Jahn et al., 2004, 2008). The vestibular cortex is part of the multisensory cortex, which is thought to process vestibular information (Guldin and Grüsser, 1998). Therefore, we hypothesized that combined cerebellar tACS and AC-GVS at the gait frequency would increase the postural response in a phase-dependent manner (in-phase or anti-phase), and that this effect would vary depending the presence or absence of imaginary walking. We first investigated the effects of AC-GVS alone on the postural response during walking imagery and no-imagery sessions with three stimulation frequency conditions. We then evaluated the effects of combined cerebellar tACS and AC-GVS, in-phase and anti-phase, with and without walking imagery.

## Materials and methods

### Participants

Thirty-two participants took part in this study. None of the participants had any history of neurological, psychiatric, or other medical problems. Written informed consent was obtained from each participant in accordance with the Declaration of Helsinki. This study was approved by the Ethics Committee of the Fukuoka International University of Health and Welfare. Sixteen participants (8 women; mean age ± standard deviation (SD): 20.8 ± 1.0 years old) completed Experiment 1. This sample size was chosen based on previous studies that reported a postural response to AC-GVS (Petersen et al., 1994; Latt et al., 2003). Based on the effect size in Experiment 1, a sample size of *N* ≥ 17 was determined to be necessary to achieve a statistical power of 0.95 in Experiment 2. Therefore, 20 participants (11 women; 21.6 ± 3.2 years old; four were also included in Experiment 1) took part in Experiment 2.

### Procedures

This study had a randomized double-blind sham-controlled design.

#### Experiment 1

We confirmed the effects of AC-GVS on the periodic postural response. The experiment included motor imagery (imagery) and no-imagery sessions (Figure 1D). Before the study began, the participants were trained to imagine movement for the motor imagery sessions. The participants were instructed to imagine the kinesthetic sensation generated by gait as vividly as possible from a first-person perspective. They did this while standing in a relaxed position with their eyes closed. In the imagery session, during which postural responses were measured, the participants were asked to imagine that they were walking in a natural way in a straight line. In the no-imagery session, they were instructed not to imagine any body movements. We assessed the effects of AC-GVS with four stimulation frequency conditions: gait frequency, gait frequency minus 0.3 Hz (slow), gait frequency plus 0.3 Hz (fast), and a sham condition (Figure 1E, left). We also measured the individual gait frequency from actual locomotion for gait analysis (Figure 1D). The postural response measurements lasted 70 s in the baseline and stimulation conditions, and the interval between the different conditions was 120 s. Each session lasted 830 s with a 5 min break in between sessions to avoid mental fatigue. The participants were instructed to stand quietly with their eyes closed during the measurements. In each session, postural measurements at baseline (no stimulation) were performed prior to the stimulation trials. For the baseline measurement in the imagery session, the postural response was evaluated without stimulation while the participant imagined movement. The orders of the stimulation conditions and sessions were randomized across participants.

#### Experiment 2

We explored the functional connectivity between the vestibular and cerebellar systems in terms of the periodic postural response. There were three stimulus conditions: in-phase (0° phase difference between left vestibular and left cerebellum stimulation), anti-phase (180° phase difference between stimulations), and sham stimulation (Figure 1E, right). The stimulation frequency was set to the individual gait frequency in all conditions. The other procedures were identical to those of Experiment 1.

### AC-GVS and tACS

Alternating current galvanic vestibular stimulation and tACS were performed using the DC Stimulator-Plus (NeuroCare Group GmbH, Munich, Germany). The stimulation waveform was sinusoidal without DC offset. AC-GVS was applied *via* self-adhesive electrodes (3 × 3 cm) (Axelgaard Manufacturing Co., Ltd., USA) over both mastoid processes (Figures 1A, B). The tACS electrode (3 × 3 cm) was centered 3 cm left-lateral from the inion, while the reference electrode (5 × 7 cm) was placed on the lower right posterior part of the neck (Figure 1B). We chose these tACS electrode positions because we sought to influence activity in the cerebellum (Koganemaru et al., 2020). The electrodes were fixed using surgical tape and a support bandage. Combined tACS and AC-GVS was controlled by an external controller (DC-StimEditor, Medical Try System, Tokyo, Japan) connected to two stimulators. The AC-GVS and tACS stimulation intensities were the same, i.e., 80% of an individually determined threshold that elicited phosphenes and skin sensations but not vestibular sensation. To reduce the electrode impedance, the skin was cleaned using alcohol and exfoliating cleanser (SkinPure; Nihon Kohden, Tokyo, Japan), and electrode gel (Gelaid; Nihon Kohden) was then applied. The impedance was kept below 10 kΩ. AC-GVS and tACS in Experiments 1 and 2 were applied for 60 s with a 5 s ramp-up and -down period. For sham stimulation, AC-GVS at gait frequency was applied for only 10 s at the beginning of the 70-s period in Experiments 1 and 2. The AC-GVS signal was recorded with a 1 kΩ resistor connected in series to the AC stimulator and stored to calculate the phase-locking value (PLV) with the postural response represented by the inertial measurement unit (IMU) sensor signal in Experiment 2.

The electrical fields of the AC-GVS or combined stimulation montages were simulated using the simNIBS pipeline (v3.2.6^1^) (Saturnino et al., 2019). A finite element head model was derived from MRI data of one subject who did not participate in this study. The following parameters were set for this computation: electrode size, 3 × 3 cm; tACS reference electrode size, 5 × 7 cm; current strength, 0.35 mA (0.7 mA peak-to-peak); electrode thickness, 1 mm; and no sponges. The electrical field of the combined stimulation was localized over the cerebellum, with a peak electric field over the left midline cerebellar structures (Figure 1C, right), while AC-GVS alone produced no obvious electric field over the cerebral cortex or the cerebellar cortex (Figure 1C, left).

### Inertial measurement unit orientation estimate

Data were acquired using the IMU system (Myomotion, Noraxon, Scottsdale, AZ, USA), which was wireless and had wearable sensors. IMU sensors were attached using elastic straps at six locations: the head (the back of the head, Figure 1A), the upper thoracic area (C7 along the spinal cord), the thoracic area (Th12 along the spinal cord), the pelvis (bony part of the sacrum), and the right and left feet. The 3D orientation angle was calculated from the 3D accelerometer, gyroscope, and magnetometer data from each sensor. The orientation of the head sensor was represented by the yaw, pitch, and roll angles, which are associated with head axial rotation, frontal bending, and lateral bending, respectively (Figure 1A). The sensor signals were recorded using MyoResearch software (Noraxon), digitized at a sampling rate of 100 Hz for the gait measurements and 1,500 Hz for the postural response measurements. For the analysis, the orientation angle at each sensor was differentiated to calculate the angular velocity.

### Gait analysis

The participants were asked to walk straight forward for 16 m at a comfortable pace. This was performed three times. During gait analysis, the heel contact points were identified according to the acceleration signal from the left foot sensor (Tanimoto et al., 2017). The stride frequency was calculated from the middle five strides. We used the averaged stride frequency of the three walking trials, with a 0.1 Hz bin, as the gait frequency.

### Data analysis

Data analyses were performed using functions implemented in Python 3.8 and the SciPy package (Virtanen et al., 2020).

**FIGURE 2:**
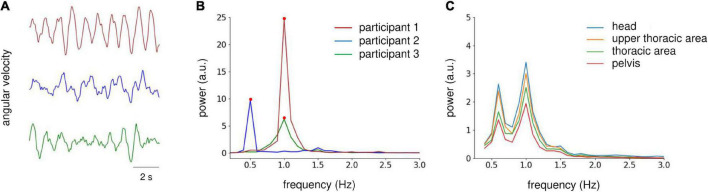
Analysis of IMU sensor data on the yaw axis. **(A)** Raw angular velocity data at the yaw axis obtained from the head sensor of three participants during AC-GVS stimulation at the gait frequency in the imagery condition in Experiment 1. **(B)** Power spectrum density (PSD) of velocity data from the three participants shown in **(A)** (illustrated by the same color). We focused on the peak power (filled circles) from 0.3 to 5 Hz. **(C)** Grand average PSD across all participants for each IMU sensor in Experiment 1. The distribution of PSD was similar for all sensors. Therefore, we examined IMU data for the head area.

During the periodic postural response analysis, the angular velocity signals were filtered using a band-pass filter (zero-lag, range 0.3–20 Hz) and down-sampled to 100 Hz. Then, the signals at the middle 60-s period were extracted and cut into six epochs, each with a length of 10 s. For the peak power analysis of actual gait, zero-padding was applied to the angular velocity data (i.e., five gait strides) for each gait trial to match the data length of the postural response analysis epoch. Then, the peak power was averaged for the three trials. We visually inspected the epochs, and those with excessive artifacts were rejected from further analysis (one epoch was excluded in total). The power spectral density (PSD) was estimated *via* fast Fourier transformation (frequency resolution, 0.1 Hz) for each epoch, and then averaged across the epochs. For the periodic postural response and gait analyses, we focused on the peak power from 0.3 to 5 Hz because the peak power existed within this range (Figures 2A, B). Then, we conducted logarithmic transformations (log10) to normalize the data distribution of the peak power. To determine the target sensor for analysis, we checked the grand average PSD for each sensor orientation in Experiment 1, which revealed similar power distribution at all sensors (Figure 2C). Therefore, we used the sensor data from the head area for further analysis.

In Experiment 2, we evaluated whether the rhythmic postural response during stimulation was synchronized with the applied AC-GVS. We used the PLV (Lachaux et al., 1999) to estimate the phase synchrony between the postural response and the AC-GVS signal. The time-averaged PLV was computed *via* the following equation:


$$P\,L\,V=\frac{1}{N}\,\left|\sum_{k=1}^{N}e^{i\,\left(\theta_{1}\,\left(k\right)-\theta_{2}\,\left(k\right)\right)}\right|$$

where *N* stands for the length of the signal and θ1 and θ2 are the instantaneous phase values (Hilbert transform, range 0.3–5 Hz) at the time point *k* of the IMU sensor signal and the stimulation signal of the left AC-GVS, respectively. The value of PLV is within [0, 1], where 0 represents a random phase relationship and 1 reflects perfect phase synchrony between the rhythmic postural response of the IMU signal and the AC-GVS signal. In the sham condition, we used a template of the AC-GVS signal for the PLV analysis.

### Statistical analysis

To test the effects of the imagery and stimulus conditions, we conducted a two-way repeated-measures analysis of variance (rmANOVA) for peak power with “imagery” (imagery and no-imagery) and “frequency” (Experiment 1: baseline, slow, gait, fast, and sham conditions) or “phase” (Experiment 2: baseline, in-phase, anti-phase, and sham conditions) as factors. The Greenhouse–Geisser correction was used when sphericity was lacking. When the interaction was significant, we used a one-way rmANOVA with the stimulus conditions as a factor in each session. We then performed *post hoc* analyses using paired *t*-tests with the Holm–Bonferroni correction for multiple comparisons. Pearson’s correlation coefficients were calculated to estimate the correlation between peak power during the combined stimulation and that during actual locomotion. Statistical analyses were carried out using R (R Core Team, 2020).

## Results

No study participant reported any adverse effects during or after the stimulation sessions. None of the participants could discriminate the stimulation from sham trials. The stimulation intensity (peak-to-peak) of the AC-GVS and tACS was as follows: Experiment 1, mean ± SD, 0.75 ± 0.16 mA (0.475–1.05 mA); Experiment 2, 0.69 ± 0.22 mA (0.24–1.12 mA). The gait frequencies were as follows: Experiment 1, 0.98 ± 0.04 Hz (0.9–1.0 Hz); Experiment 2, 1.0 ± 0.08 Hz (0.8–1.1 Hz).

**FIGURE 3:**
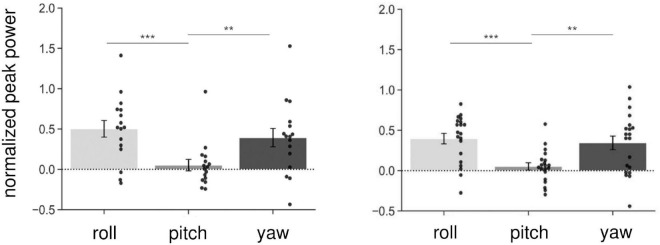
Normalized peak power on the three axes in Experiment 1 (left) and Experiment 2 (right). As it was normalized, the peak power of the baseline was subtracted from that in the stimulus conditions (Experiment 1: slow, gait, and fast conditions; Experiment 2: in-phase and anti-phase conditions), and then collapsed over stimulations and sessions for each axis. Each dot indicates peak power on each axis for one participant. AC-GVS or combined stimulation increased peak power on the roll and yaw axes compared with the pitch axis. Error bars indicate the standard error of the mean (SEM). **p < 0.01, ***p < 0.001.

We first investigated whether AC-GVS alone or combined stimulation induced a periodic postural response, and determined the axis of movement. We subtracted the peak power at the baseline from that in the stimulation conditions (Experiment 1: slow, gait, and fast conditions; Experiment 2: anti-phase and in-phase conditions) and then collapsed the data for each axis. As shown in Figure 3, AC-GVS (Experiment 1: left) and combined stimulation (Experiment 2: right) evoked rhythmic postural responses on the roll and yaw axes but not the pitch axis. A one-way rmANOVA revealed significant effects of “axis” in Experiments 1 and 2 (Experiment 1: *F*_(2,30)_ = 15.722, *p* < 0.001, *η_*p*_*^2^ = 0.512; Experiment 2: *F*_(2,38)_ = 10.292, *p* < 0.001, *η_*p*_*^2^ = 0.351). *Post hoc* comparison revealed that AC-GVS and combined stimulation increased the peak powers in the roll and yaw directions compared with that in the pitch direction (Experiment 1, roll vs. pitch: *p* < 0.001; yaw vs. pitch: *p* = 0.006; roll vs. yaw: *p* = 0.215; Experiment 2, roll vs. pitch: *p* < 0.001; yaw vs. pitch: *p* = 0.009; roll vs. yaw: *p* = 0.561). Therefore, we analyzed the postural responses on the roll and yaw axes. The peak frequencies of the periodic postural responses on the roll and yaw axes are summarized in Table 1.

**TABLE 1:** Peak frequencies of periodic postural response on the roll and yaw axes in each condition in Experiments 1 and 2 (mean ± SD).

	Experiment 1					Experiment 2			
	Baseline	Slow	Gait	Fast	Sham	Baseline	Anti	In	Sham
Roll	1.2 ± 0.6	0.7 ± 0.1	1.0 ± 0.3	1.1 ± 0.2	1.0 ± 0.3	1.6 ± 0.8	1.1 ± 0.4	1.1 ± 0.5	1.3 ± 0.6
Yaw	1.2 ± 0.3	0.8 ± 0.3	1.0 ± 0.2	1.0 ± 0.2	1.0 ± 0.4	1.3 ± 0.3	1.1 ± 0.3	1.0 ± 0.3	1.2 ± 0.4

## Experiment 1

### The effects of AC-GVS on peak power

**FIGURE 4:**
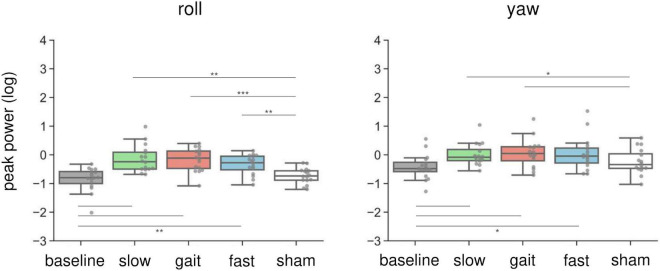
Effects of AC-GVS on peak power in each motion direction (roll, left; yaw, right) in Experiment 1. Peak power data were collapsed over the imagery and no-imagery sessions. Box plots show the 25th to 75th percentile, with the median shown as a horizontal line. Each dot indicates the peak power in each stimulation condition for one participant. AC-GVS at all frequencies increased the roll peak power compared with the baseline and sham conditions, while the yaw peak power was enhanced by AC-GVS at the slow and gait frequencies. *p < 0.05, **p < 0.01, ***p < 0.001.

Figure 4 illustrates the changes in peak power of the head sensor in the roll and yaw orientations. In brief, AC-GVS at all frequencies induced a periodic postural response on the roll axis, and AC-GVS in the slow and gait conditions increased the yaw peak power, compared with the sham condition, irrespective of imagery. For the peak power of head roll motion, a two-way rmANOVA showed a significant effect of “frequency” (*F*_(4,60)_ = 17.25, *p* < 0.001, *η_*p*_*^2^ = 0.535), but no significant effects of “imagery” (*F*_(1,15)_ = 4.095, *p* = 0.061, *η_*p*_*^2^ = 0.214) or “frequency” × “imagery” interaction (*F*_(4,60)_ = 0.8, *p* = 0.53, *η_*p*_*^2^ = 0.051). A *post hoc* comparison of the main effect of “frequency” showed that AC-GVS in all frequency conditions significantly increased the postural response compared with the baseline and sham conditions (*p* ≤ 0.003), while there were no significant differences between stimulation conditions (i.e., slow, gait, and fast) (*p* ≥ 0.485). A two-way rmANOVA for the peak power of head movement on the yaw axis revealed significant effects of “imagery” (*F*_(1,15)_ = 17.926, *p* = 0.001, *η_*p*_*^2^ = 0.544) and “frequency” (*F*_(2.63,39.44)_ = 5.96, *p* = 0.003, *η_*p*_*^2^ = 0.284), but no significant “frequency” × “imagery” interaction (*F*_(2.14,32.08)_ = 2.619, *p* = 0.085, *η_*p*_*^2^ = 0.149). *Post hoc* comparison of the main effect of “frequency” indicated that AC-GVS in the slow and gait conditions led to increased peak power compared with the baseline and sham conditions (*p* ≤ 0.036). However, there was no significant difference between the fast and sham conditions (*p* = 0.244).

## Experiment 2

### The effects of combined stimulation on peak power

**FIGURE 5:**
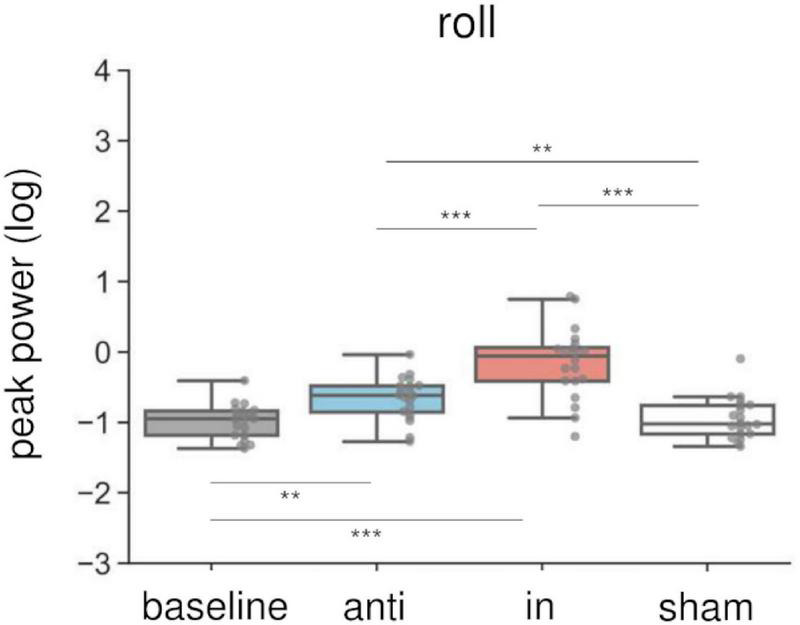
Effects of combined AC-GVS and tACS on roll peak power in Experiment 2. The peak power data were collapsed across sessions in each stimulation condition. Each dot shows individual peak power. Combined in-phase stimulation facilitated the roll peak power compared with the baseline, sham, and anti-phase conditions. **p < 0.01, ***p < 0.001.

**FIGURE 6:**
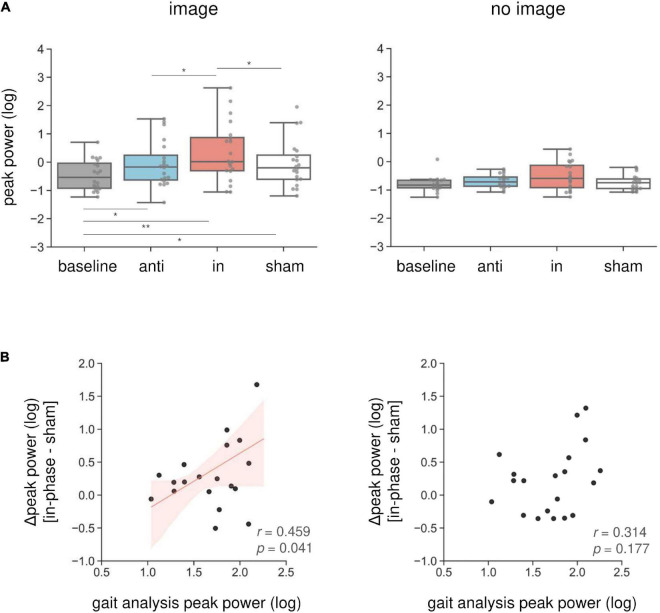
Imagery-specific effects on the yaw axis. **(A)** Effects of combined stimulation on the yaw peak power in the imagery (left) and no-imagery (right) sessions. In-phase stimulation increased the yaw peak power compared with the other conditions, while there were no differences in the no-imagery session. **(B)** The relationship between in-phase stimulation and actual locomotion on the yaw axis. The peak power in the sham condition was subtracted from the in-phase stimulation peak power to calculate the Δ peak power. Correlations between the yaw peak power during gait and the Δ peak power during in-phase stimulation were calculated in the imagery (left) and no-imagery (right) sessions. Positive correlations were only observed in the imagery session. The regression lines are shown in red. The shaded areas reflect the 95% confidence interval. *p < 0.05, **p < 0.01.

Combined in-phase cerebellar tACS and AC-GVS-induced rhythmic postural responses on the roll axis irrespective of imagery (Figure 5). A two-way rmANOVA revealed a significant main effect of “phase” (*F*_(1.9,36.09)_ = 36.891, *p* < 0.001, *η_*p*_*^2^ = 0.66), but no significant effects of “imagery” or “phase” × “imagery” interaction (*p* ≥ 0.559). A *post hoc* analysis of “phase” revealed greater peak power in the in-phase condition compared with the other conditions (*p* ≤ 0.001). In contrast, yaw motion was increased by in-phase stimulation during the imagery but not the no-imagery trials (Figure 6A). A two-way rmANOVA showed significant effects of “phase” (*F*_(2.23,42.41)_ = 12.271, *p* < 0.001, *η_*p*_*^2^ = 0.392) and “imagery” (*F*_(1,19)_ = 13.715, *p* = 0.002, *η_*p*_*^2^ = 0.419). The interaction between “phase” and “imagery” was also significant (*F*_(3,57)_ = 3.763, *p* = 0.016, *η_*p*_*^2^ = 0.165). We performed a further one-way rmANOVA for each trial to identify the stimulation conditions with imagery-dependent effects. We found significant “phase” effects in the imagery trials (*F*_(3,57)_ = 10.349, *p* < 0.001, *η_*p*_*^2^ = 0.353), and *post-hoc* tests indicated that the periodic postural response was greater in the in-phase condition compared with the other conditions (*p* ≤ 0.04) (Figure 6A, left). Although a one-way ANOVA revealed a significant effect of “phase” in the no-imagery sessions (*F*_(1.88,35.76)_ = 3.462, *p* = 0.045, *η_*p*_*^2^ = 0.154), *post hoc* analyses showed no significant differences between any of the conditions (*p* ≥ 0.125), unlike that observed for the imagery trials (Figure 6A, right).

We found an imagery-dependent effect of in-phase stimulation on yaw peak power. Therefore, we calculated the correlation coefficients between the yaw peak power during in-phase stimulation and that of actual locomotion recorded during gait analysis to investigate the relationship between in-phase effects and gait. We subtracted the peak power in the sham condition from that in the in-phase condition, producing the Δ peak power. The Δ peak power in the imagery trials was positively correlated with the peak power during gait measurements (*r* = 0.46, *p* = 0.041) (Figure 6B, left). This correlation was not present for the no-imagery trials (*r* = 0.314, *p* = 0.177) (Figure 6B, right).

### The effects of combined stimulation on PLV

**FIGURE 7:**
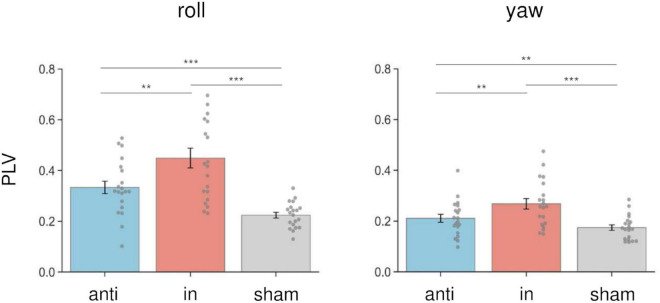
Effects of combined stimulation on PLV in the roll (left) and yaw (right) directions in Experiment 2. The PLV data were collapsed across sessions in each stimulation condition. In-phase stimulation increased the PLV in the roll and yaw directions compared with the sham and anti-phase conditions. Error bars indicate SEM. **p < 0.01, ***p < 0.001.

We calculated the PLV between the postural response and the left AC-GVS signal to estimate whether the periodic postural response was synchronized to the AC-GVS (and tACS) in Experiment 2. In terms of head roll motion, a two-way rmANOVA indicated a significant “phase” effect (*F*_(1.43,27.15)_ = 30.947, *p* < 0.001, *η_*p*_*^2^ = 0.62), but no significant effects of “imagery” (*F*_(1,19)_ = 0.916, *p* = 0.351, *η_*p*_*^2^ = 0.046) or “phase” × “imagery” interaction (*F*_(2,38)_ = 0.303, *p* = 0.74, *η_*p*_*^2^ = 0.016). *Post hoc* analyses for the effect of “phase” revealed increased PLV in the in-phase condition compared with the anti-phase and sham conditions (*p* ≤ 0.001) (Figure 7, left). In terms of yaw motion, a two-way rmANOVA showed a significant “phase” effect (*F*_(2,38)_ = 25.104, *p* < 0.001, *η_*p*_*^2^ = 0.569). However, the effects of “imagery” (*F*_(1,19)_ = 1.767, *p* = 0.2, *η_*p*_*^2^ = 0.085) and the “phase” × “imagery” interaction (*F*_(2,38)_ = 0.888, *p* = 0.42, *η_*p*_*^2^ = 0.045) were not significant. *Post hoc* analysis for the effect of “phase” revealed increased PLV in the in-phase stimulation condition compared with the other conditions (*p* ≤ 0.002) (Figure 7, right).

## Discussion

To our knowledge, this study is the first to report modulation of the vestibular–cerebellar network in terms of the posture and locomotor systems using combined tACS and AC-GVS. Combined in-phase stimulation led to increased yaw peak power compared with the baseline, sham, and anti-phase conditions during walking imagery only. This imagery-dependent effect was not found for AC-GVS alone in Experiment 1. Moreover, the yaw peak power induced by in-phase stimulation in the imagery session was positively correlated with that during actual gait. In contrast, the roll power was enhanced in the in-phase stimulation condition for both the imagery and no-imagery trials, and AC-GVS alone had similar effects to those of combined stimulation. PLV analysis also suggested that in-phase stimulation-induced roll and yaw motions were synchronized to the AC-GVS signal regardless of the imagery condition. Therefore, combined stimulation appeared to differentially affect head motion on the roll and yaw axes depending on walking imagery, indicating that the vestibular–cerebellar network is implicated in both postural control and locomotion systems.

### The effects of AC-GVS on postural control

Alternating current galvanic vestibular stimulation evokes sinusoidal head motion (Dlugaiczyk et al., 2019) and torsional eye movements (Kwan et al., 2019). Here, AC-GVS at slow and gait frequencies increased the peak power of head movements on the roll and yaw axes in Experiment 1, indicating that the stimulation induced periodic sway of lateral bending and head rotation. Indeed, previous studies reported that bipolar GVS to both mastoids evoked postural responses in the roll and yaw directions (Fitzpatrick and Day, 2004; Aoyama et al., 2015). In vestibular electrophysiology studies, GVS stimulated the spike trigger zone on the primary vestibular afferents (Fitzpatrick and Day, 2004). Moreover, GVS evoked motor responses and action potentials in the vestibulospinal tract in an animal study (Muto et al., 1995), and AC-GVS activated both canal and otolith afferent populations (Kwan et al., 2019). Together, these AC-GVS effects support the role of the vestibular system in postural control.

The effects of AC-GVS on postural control are influenced by stimulation frequency and intensity (Coats, 1972; Petersen et al., 1994, 1995; Latt et al., 2003). We found no significant differences in yaw motion between the fast and sham conditions in the present study. Animal studies have shown that AC-GVS at frequencies above 1 Hz can increase the phase lead of GVS-evoked spike discharges from vestibular afferents (Goldberg et al., 1982; Ezure et al., 1983). This phase lead is likely caused by the direct activation of vestibular afferents, as the signal bypasses canal fluid dynamics, ciliary bundle deflection, and synaptic transmission between hair cells and afferent fibers (Dlugaiczyk et al., 2019). Given that the gait frequency in our study population was around 1 Hz, these mechanisms might explain the weak effects of AC-GVS in the fast frequency trials.

### The influence of vestibular–cerebellar network on postural-gait control

In Experiment 1, although AC-GVS at all frequencies induced periodic postural responses, AC-GVS effects were not influenced by gait imagery. This could be interpreted to mean that the vestibular system and locomotor system are not directly linked. However, direct current or noise current GVS was found to affect postural performance (Iwasaki et al., 2014; Goel et al., 2015; Inukai et al., 2018) and locomotor performance (Bent et al., 2004; Wuehr et al., 2016a,b; Iwasaki et al., 2018). Many vestibular primary afferents project to the ipsilateral cerebellum as mossy fibers (Barmack and Yakhnitsa, 2003), and the cerebellum is known to play a crucial role in regulating locomotion (Powell et al., 2015). In animal studies, the cerebellum was found to drive the spinal locomotor circuits *via* its rhythmic bursts, resulting in locomotion (Mori et al., 1999). Moreover, the cerebellum provides locomotor rhythm onto vestibulospinal neurons (Orlovsky, 1972; Witts and Murray, 2019). In humans, the vestibular–cerebellar interaction was investigated by non-invasive brain stimulation using transcranial magnetic stimulation. Continuous theta burst stimulation induced inhibitory effects in the cerebellum after stimulation, and then modulated the vestibular muscle response induced by square wave pulse GVS (Lam et al., 2017). Moreover, the effects of noisy GVS on vestibular function were facilitated after low-frequency repetitive transcranial magnetic stimulation over the cerebellum (Matsugi et al., 2020). These results suggest that the cerebellum modulates vestibulospinal function *via* the vestibular–cerebellar network. However, the role of the vestibular–cerebellar network in generation of locomotor patterns in humans is unclear. Accordingly, we examined the influence of the vestibular–cerebellar network on postural-gait control using combined cerebellar tACS and AC-GVS. As a result, combined stimulation at the in-phase increased the yaw peak power compared with the other conditions with but not without walking imagery. Moreover, the effect of in-phase stimulation was positively correlated with the yaw peak power during actual gait. These results suggest that the vestibular–cerebellar network modulates the locomotion system.

Previous fMRI studies have indicated that brain regions related to locomotion are activated during mental imagery of walking (Jahn et al., 2004, 2008). These studies also reported that walking imagery was associated with activation in the cerebellum. In contrast, the vestibular cortex was activated during standing imagery but not when imagining walking or running. In line with previous findings, the combined stimulation in the present study had gait imagery-dependent effects, although this was not the case for AC-GVS alone. However, the reason why the peak power modulation by in-phase stimulation was limited to the yaw axis remains unknown. Axial rotation during walking is related to walking velocity (Van Emmerik and Wagenaar, 1996), arm swing (Pontzer et al., 2009), stride length, frequency (Wagenaar and Beek, 1992), and locomotor cost (Chapman and Ralston, 1964). Additionally, yaw axis movements affected gait stability in both healthy controls and cerebellar ataxia patients (Conte et al., 2014). Thus, yaw motion during gait might contribute to gait pattern efficiency, and might be controlled by the vestibular–cerebellar network. Alternatively, since bipolar GVS to both mastoids evoked postural responses in a large roll and small yaw motion (Fitzpatrick and Day, 2004; Aoyama et al., 2015), the lack of an effect of imagery on the roll axis during in-phase stimulation could be explained by a ceiling effect. As with the modulation of roll peak power, the PLV analysis revealed no imagery-dependent effects, although in-phase stimulation increased the PLV during roll and yaw motions. Thus, in-phase stimulation evoked periodic postural responses that partly synchronized with the AC-GVS signal, regardless of imagery condition. Given these data, we propose that power modulation in a specific axis could reflect the gait-dependent effects of in-phase stimulation, while roll peak power and PLV could reflect postural control systems.

### The effects of “alternating current stimulation”

Electrical current waveforms for GVS are generally applied as a direct current, trains of short pulses, and band-limited noise. In particular, noisy GVS was effective in improving locomotion performance in both healthy participants and patients with bilateral vestibulopathy (Wuehr et al., 2016a; Iwasaki et al., 2018; Ko et al., 2020; Chen et al., 2021). The mechanism underlying these effects of noisy GVS involves stochastic resonance, which refers to the phenomenon whereby a weak (i.e., sub-threshold) signal is amplified by adding noise (Iwasaki et al., 2014). By contrast, the mechanism underlying the effects of alternating current stimulation was suggested to involve entrainment of ongoing brain oscillations. Our findings suggest that combined cerebellar tACS and AC-GVS can uncover the fine-grained temporal dynamics of the vestibular–cerebellar network, as shown by the phase dependence. Thus, combined stimulation using alternating current targets endogenous network dynamics, and it is different from other GVS approaches.

Transcranial alternating current stimulation can entrain brain oscillations and modulate brain functions in a frequency-dependent manner (Herrmann et al., 2013; Nakazono et al., 2020; Vogeti et al., 2022). Phase-dependent effects of tACS have also been found in motor (Goldsworthy et al., 2016; Guerra et al., 2016; Nakazono et al., 2016, 2021; Schilberg et al., 2018; Ogata et al., 2021), auditory (Neuling et al., 2012; Riecke et al., 2015), and visual function (Helfrich et al., 2014). In the locomotor system, tACS at the gait frequency over the cerebellum synchronized the natural walking rhythm (Koganemaru et al., 2020). Thus, tACS appears to entrain neuronal activities related to gait generation in the cerebellar system. In rats, a low-frequency alternating current applied over the cerebellum induced burst-like activity of Purkinje cells (Asan et al., 2020). tACS has been used to investigate long-range functional connectivity in the brain (Polanía et al., 2012; Strüber et al., 2014; Violante et al., 2017; Schwab et al., 2019; Salamanga-Giron et al., 2021). The functional connectivity revealed by tACS has been supported by fMRI (Violante et al., 2017), electroencephalography (EEG) (Helfrich et al., 2016), and intracranial EEG (iEEG) (Alagapan et al., 2019) studies. Regarding cortical modulation by GVS, the brain regions in the cortical vestibular network are widely distributed, and the parieto-insular vestibular cortex (PIVC) has been proposed as the core vestibular cortex (Lopez and Blanke, 2011). In fMRI studies, 1-Hz AC-GVS indicated that the temporo-parietal junction was the PIVC (Lobel et al., 1998). Moreover, Stephan et al. (2005) reported that 1-Hz AC-GVS activated the insula, superior temporal gyrus, and cerebellum. Accordingly, the regions of the vestibular–cerebellar network modulated by combined stimulation might include the vestibular cortex, such as the PIVC. Future studies are required to explore the influence of combined stimulation on oscillatory brain networks.

### Limitations

Our study had several limitations. First, we examined the effects of combined stimulation on imagined walking but not actual locomotion to avoid confusion between stimulation effects and the complex sensory integration induced by locomotion in the vestibular–cerebellar network. Evaluating gait performance during actual walking makes it difficult to determine whether the combined stimulation affects generation of the locomotor pattern or simply maintains balance and upright posture during walking. In the present study, there are no differences in the postural responses required during the image and no-image conditions, although we consider there are differences in the activated brain network. Moreover, because AC-GVS induces a periodic posture response, it is difficult to identify between gait-induced and GVS-induced oscillatory head motion. For these reasons, we evaluated the effects of combined stimulation in the imagery and no-imagery sessions. However, imagined locomotion differs from actual locomotion in that correlated sensory input from proprioceptive, vestibular, and visual systems is absent (Jahn et al., 2008). Understanding the effects of combined stimulation on gait performance will be the next challenge. Second, we did not examine the effects of cerebellar tACS alone on postural response. This was because we ran preliminary tests and found that tACS alone induced no postural responses. Thus, cerebellar tACS alone would not explain the effects of in-phase stimulation. Finally, the current paths might differ between two-pole (AC-GVS) and three-pole (combined stimulation). A previous study revealed that different current paths in the vestibular organ could generate directional differences in head motion (Aoyama et al., 2015). However, in the current study, the head motion patterns were similar between the AC-GVS and combined stimulation conditions (Figure 3). Furthermore, the imagery-dependent effect of in-phase stimulation in Experiment 2 cannot be explained by differences in the current paths in the vestibular organ.

## Conclusion

Using the IMU sensor, we provide new evidence that combined in-phase cerebellar tACS and AC-GVS increase the periodic postural responses on the yaw axis during walking imagery only. The effects of in-phase stimulation on the yaw axis correlated with the yaw motion of actual gait. By contrast, these imagery-dependent effects were not observed with AC-GVS alone. Therefore, our results suggest that the vestibular–cerebellar network plays a role in posture-gait control. These findings may contribute to the development of therapeutic applications using combined stimulation.

## Data availability statement

The raw data supporting the conclusions of this article will be made available by the authors, without undue reservation.

## Ethics statement

The studies involving human participants were reviewed and approved by the Ethics Committee of the Fukuoka International University of Health and Welfare. The patients/participants provided their written informed consent to participate in this study.

## Author contributions

HN, TT, TM, and KO conceived the project and designed the experiment. HN, TT, and TM performed the data collection. HN conducted the experiment. HN, AT, EY, and KO analyzed the data. HN and KO wrote the first draft of the manuscript. All authors contributed to the final draft of the manuscript and approved the submitted version.

## Footnotes

1
www.simnibs.org
